# To block or not to block—hormonal signaling in the treatment of cancers

**DOI:** 10.3389/fendo.2023.1129332

**Published:** 2023-02-20

**Authors:** Apoorva Abikar, Chriswin Saimon, Prathibha Ranganathan

**Affiliations:** Centre for Human Genetics, Bengaluru, India

**Keywords:** endocrine therapies, steroid hormones, tumor microenvironment, cell specific effects, mouse models, altered metabolism

## Abstract

The breast and prostate glands are the two major organs that are highly dependent on the gonadal steroid hormones for their development and homeostasis. The cancers of these organs also show a large dependence on steroid hormones and have formed the basis of endocrine therapy. Estrogen deprivation by oophorectomy has been in active practice since the 1970s, and androgen deprivation therapy for prostate cancer was a major breakthrough in medicine in 1941. Since then, several improvisations have happened in these modes of therapy. However, the development of resistance to this deprivation and the emergence of hormone independence are major problems in both cancers. The lessons learned from rodent models have made it clear that the male hormone has a role in females and *vice versa*. Also, the metabolic products of these hormones may have unintentional effects including proliferative conditions in both sexes. Hence, administering estrogen as a method of chemical castration in males and administering DHT in females may not be the ideal scenario. It would be important to consider the status of the opposite sex hormone signaling and its effects and come up with a combinatorial regime to strike a balance between androgen and estrogen signaling. This review summarizes the current understanding and developments in this field in the context of prostate cancer.

Nuclear hormone receptors are intracellular receptors that are activated by lipid-soluble ligands such as steroid hormones. Once bound by the ligand, most receptors function as transcription factors to control gene expression affecting numerous biological processes (reviewed in (1)). Steroid hormones are derivatives of cholesterol and have a wide variety of physiological functions. These are classified based on the site of synthesis. The hormones which are synthesized by the gonads, both in males and females, are called gonadal steroids or sex hormones. Gonadal steroid hormone synthesis is mainly regulated by the hypothalamic–pituitary–gonadal (HPG) axis. The release of gonadotropin-releasing hormone (GnRH) from the hypothalamus results in the release of luteinizing hormone (LH) and follicle-stimulating hormone (FSH) from the pituitary into general circulation. The binding of LH to its receptors on Leydig cells in males and on theca cells in females causes the enhanced expression of the steroidogenic acute regulatory protein (StAR). This protein helps cholesterol to move to the inner membrane of the mitochondria, where it is converted into pregnenolone by the cytochrome p450scc (CYP11A1) enzyme. Pregnenolone acts as a precursor for both androgen and estrogen in both males and females (2). **Figure S1** summarizes the biosynthesis of these steroid hormones in both males and females.

Nuclear hormone signaling occurs when the ligand (usually a steroid derivative such as estrogen and androgen) diffuses into the cell and binds to the receptor. The receptors dimerize and translocate to the nucleus and function as transcription factors. Along with other co-activators or co-repressors, they bring about changes in gene transcription [reviewed in (1)]. All the nuclear hormone receptors share a similar domain architecture, and the mode of signaling and activation is also similar. In the case of estrogen receptor (ER) and androgen receptor (AR) signaling, the signaling can occur by canonical/genomic signaling or non-canonical/non-genomic signaling. The non-canonical signaling can be activated either by variant receptors or membrane-bound receptors and sometimes by a cross talk with other pathways [reviewed in (3)] (**Figure 1**). These non-canonical or non-genomic actions are likely to play a role in pathophysiological processes including proliferative, inflammatory, and immune-related diseases (4, 5). It is also possible that these mechanisms may also have a role in resistance to endocrine therapies (3). Since the mode of signaling is very similar for the AR and ER, this could be applicable to both AR- and ER-targeted therapies, which are relevant in prostate and breast cancers, respectively (6).

**Figure 1:**
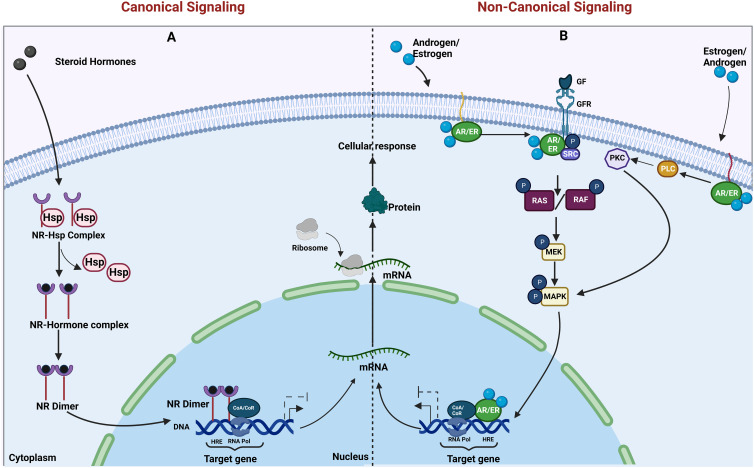
Canonical and non-canonical actions of nuclear hormone receptors. **(A)** The genomic and **(B)** non-genomic actions of steroid hormone receptors (AR and ER) are illustrated. **(A)** Canonical actions require the binding of steroid hormones to its receptors in the cytoplasm, resulting in the release of heat shock proteins (Hsps) from the nuclear receptor (NR)–Hsp complex, followed by dimerization of the nuclear receptors. The dimer translocates to the nucleus and regulates transcription upon binding to the hormone response element (HRE) on target gene promoters. **(B)** In non-canonical actions, the membrane-tethered steroid hormone receptors [androgen receptor/estrogen receptor (AR/ER)] either bind to growth factor receptors and activate their action or activate the enzyme cascade resulting in the activation of proliferative pathways and transcription of their downstream target genes. Created in Biorender.com.

Androgens such as testosterone and dihydrotestosterone are known to control sexual characteristics in males. Similarly, estrogen, the steroid hormone produced mainly in the granulosa and theca cells of the ovaries along with its production in the corpus luteum, is responsible for the development of female sexual characteristics. Although androgens and estrogens are commonly known as the male and female sex hormones, respectively, the physiological roles of these gonadal steroids in the opposite sex have become evident mostly due to the insights gained from rodent models, particularly from estrogen receptor knockout (ERKO) and androgen receptor knockout (ARKO) mice models.

Estrogen receptors ER-α and ER-β are coded by two genes, namely, *ESR1* (estrogen receptor 1) and *ESR2* (estrogen receptor 2), respectively. When these genes are knocked out in mouse models either alone or in combination, the effect is seen in both in the males and females. Both males and females are infertile in the case of ER-α knockout. In females, there are severe defects in the uterus such as insensitivity to estradiol and no implantation. The mammary gland development is also severely affected [(7) and references therein]. There is a reduction in ductal morphogenesis or alveolar development (8). ER-β knockout shows normal mammary development (8).

In the male ER-α knockout mouse, although the testes show normal development, there is a decrease in the weight of the testes with age, retention of fluid and dilation of seminiferous tubules, and poor motility of sperms (7). However, the male ER-β knockout mouse is fertile but with increased Leydig cells and decreased germ cells resulting from germ cell apoptosis (9). The double knockout (*ESR1*/*ESR2*) shows similar features as *ESR1* knockout, suggesting a dominant role for *ESR1* and associated signaling (9).

Although the effect of *ESR1* knockout on the male reproductive organs and function is described well, its effect on the prostate is not so well understood. Few previous studies have shown how prostatic epithelial tissue depends on estrogen for its differentiation. Estrogen also has been shown to have an effect on prostatic angiogenesis (10, 11). The ER-α and ER-β are expressed in the prostate of many species, but the knockouts in mice show no major defects in development (7). However, estrogens do play an important role in the growth, differentiation, and homeostasis of normal prostate and also in prostate carcinogenesis (11). Also, there are differential effects of the ER-α and ER-β in the initiation and progression of disease (12).

The androgen receptor is coded for by the gene *NR3C4* (nuclear receptor subfamily 3, group C, member 4). Inactivation of the androgen receptor gene in males results in the disruption of spermatogenesis by interfering with meiosis, resulting in terminating mature sperm production, causing male sterility. Meanwhile, in females, inactivation of the androgen signaling results in impairment in the health of the follicle, development and ovulation *via* intraovarian and neuroendocrine mechanisms, and female fertility (13).

Also, in females, a balance between estrogen stimulatory action and androgen inhibitory action is a key regulator in controlling cell proliferation in both normal and cancerous mammary tissues (9, 14). The human prostate is comprised of glandular epithelium with acini and ducts lined by luminal, basal, and neuroendocrine cells (15). Among these, luminal cells express the androgen receptor and respond to androgens in the body. The role of androgens in the development and maintenance of the prostate has been established quite well. However, the role seems more complex in the context of prostate cancer (16, 17).

Several mouse models have given us insight into the roles of androgen receptor and signaling in the prostate (18). While the global ARKO shows no prostate gland at all (13), the AR knocked out in specific cell types such as the epithelium and the stroma sheds light on the interplay between these cells. PEARKO, which is a model in which the AR DNA-binding function is deficient in the epithelia of the prostate, epididymis, and vas deferens, develops a normal prostate. However, later on, there is a lobe-specific reduction in prostate weight, particularly in the dorsal lobe. Increased proliferation and abnormal lesions are seen in the anterior lobe along with hindered structural and functional differentiation (19). This phenotype could be rescued when a constitutive receptor T857A transgenic was crossed with the epithelial-specific ARKO, confirming that the AR regulates growth by suppressing epithelial proliferation (20).

In intact males, it has been observed that ER-α expression is low, and an increase is seen in the prostatic epithelia in castrated animals. However, PEARKO mice show increased expression of the ER-α, as well as increased sensitivity to estrogens, which can result in increased prostate growth and squamous metaplasia. This suggests that ER expression and sensitivity to estrogens are controlled by AR-dependent mechanisms and imbalances in these may cause unwarranted effects by estrogens (21).

There are multiple mouse models where there is a stromal-specific lack of the AR (SM-ARKO). In the SM-ARKO, where the AR is knocked out in the smooth muscle cells, the adult exhibits reduced prostate weight and histological abnormalities such as hyperplasia, inflammation, fibrosis, and reduction in the epithelial, smooth muscle, and stem cell markers. Also, there are an increased sensitivity to estradiol in terms of increase in prostate weight and a decreased response to castration. These would have possible implications in prostate cancer and castrate resistance (22). Another model of SM-ARKO exhibited a reduced expression of insulin-like growth factor 1 (IGF-1) which may have an implication on the proliferation (23). FSP-ARKO is another model in which the AR is specifically knocked out in the stromal fibroblasts. This model showed decreased epithelial proliferation, increased apoptosis, and decreased collagen composition in the prostate. These observations emphasize the importance of AR signaling in the stroma and open the possibility of targeting the stromal AR in prostate cancer and hyperplasia (24).

Recent studies on two different mouse models, namely, the Hi-Myc genetic model and the T + E2 hormonal carcinogenesis model, demonstrate that stromal AR normally inhibits prostate cancer progression by restraining secretory luminal cells. This may result in unintended negative effects of androgen deprivation therapy and possibly progression toward the emergence of androgen-independent prostate cancer (25). The abovementioned observations on rodent models suggest that the AR and associated signaling have cell-type-specific and very often opposing roles in proliferation. Hence, it becomes very important to keep the context in consideration before designing therapeutic strategies.

Furthermore, from the different models of ERKO, it is clear that estrogen signaling has an important role in males, including for fertility. However, the role of estrogens on prostate development is not very clear.

The breast and prostate are two highly hormone-dependent organs, and hence, cancers arising in these organs are candidates for endocrine therapy. Oophorectomy as a treatment for breast cancer was practiced even in the 19^th^ century (26). George Beatson, a surgeon, tried ovarian ablation as a mode of treatment for inoperable cancers of the breast (27). In the 1940s, Alexander Haddow studied the effect of synthetic estrogens on breast cancer (and other cancers as well) and found that the compounds showed partial reduction in tumor growth with no evidence of the prevention of metastases and relapse (28). However, today, endocrine therapies are one of the widely used options for ER-positive cancers. The strategies used include estrogen ablation and administration of selective estrogen receptor modulators (SERMs) such as tamoxifen or aromatase inhibitors and occasionally GnRH modulators (29). These strategies not only come with side effects such as bone loss but also result in resistance very often.

Similarly, in the case of prostate, androgen deprivation therapy has been a treatment of choice (30). This is achieved by surgical or chemical castration. The latter includes 1) GnRH agonists/analogs or LHRH agonists/analogs [e.g., goserelin (Zoladex, AstraZeneca UK Limited)], 2) GnRH antagonists or LHRH antagonists [e.g., degarelix (Firmagon, Ferring Pharmaceuticals, Switzerland)], 3) Androgen receptor antagonists [e.g., flutamide and bicalutamide as first-generation drugs (Casodex, AstraZeneca UK Limited) and enzalutamide (Xtandi, Pfizer Inc. and Astellas Pharma Inc) as a second-generation drug], 4) Androgen synthesis inhibitors [abiraterone (Yonsa, Sun Pharma; Zytiga, Centocor Ortho Biotech Inc.)], and 5) Estrogens (29). In this case, like in breast cancer, resistance to ADT is a major challenge faced in treating the disease.

Treatment with estrogens is one of the modes of chemical castration used in prostate cancer treatment. It has been observed in Noble rats that prostate cancer develops when treated with estradiol and testosterone. When estrogen is administered, it gets converted into 4-catechol estrogen by a specific p450 cytochrome enzyme and oxidizes to catechol estrogen-3,4-quinones. These react with DNA and form depurinating adducts. These activate error-prone base excision repair resulting in an apurinic site eventually leading to mutations, which could be carcinogenic (31–35).

In the cell culture models of prostate cancer, it has been observed that local steroid metabolism is different in androgen-sensitive *vs*. Androgen-resistant cells (36). In LNCaP prostate cancer cells, as they progress toward an androgen refractory state, there are a decrease in oxidative activity and an increase in the reductive activity of 17-beta hydroxysteroid dehydrogenase 3 (17-βHSD). This results in the accumulation of bioactive estrogen (estradiol) in androgen refractory cells. Meanwhile, androgen-sensitive cells show a predominance of oxidized estrogens such as estrone (36).

Androgen-responsive LNCaP cells produce bioactive DHT and its derivatives as well as estrogen, while androgen-insensitive PC3 cells mainly produce oxidized androgen and estrogen derivatives such as androstenedione and estrone. This observation of altered steroid metabolism is crucial to the biological impact on the target cells (37).

The other very important factor that determines the balance between androgens and estrogens in the local tissue environment is the aromatase enzyme. Aromatase is the enzyme responsible for the conversion of androgens to estrogens and, hence, determines the local estrogen biosynthesis. It is important for regulating the balance between androgens and estrogens both at the tissue and plasma levels. Aberrant aromatase production and activity have been shown to have an important role in breast cancer. However, from the insights gained by mouse models such as ERKO, it is clear that the prostate is one of the major target organs for estrogen action. Hence, aromatase function is one of the important factors to be considered in prostate disease.

Aromatase is an enzyme coded by the gene *CYP19* (aromatase cytochrome p450). Aromatase knockout mice have been generated and have been very useful in gaining insight into the roles of the aromatase enzyme in both sexes (38–40). The phenotype of these mice includes infertility and lack of sexual behavior in both sexes, defects in folliculogenesis and spermatogenesis, increased gonadotropins and testosterone, loss of bone mass, metabolic syndrome, and insulin resistance (41).

In the prostate gland, aromatase is expressed within the stroma of benign tissue, while in cancer, there is an alternate promoter utilization leading to the induction of epithelial expression. However, the role of aromatase in the prostate and its aberrant expression and contribution to prostate carcinogenesis remains unclear. Mouse models lacking aromatase (ArKO) and overexpressing aromatase (AROM+) have given us some insights into the role of this enzyme in prostate homeostasis and disease. In aromatase knockout mice (ArKO), the T:E ratio is altered. In the absence of conversion of testosterone to estrogen, testosterone accumulates, leading to hypertrophy and hyperplasia but no malignancy. In contrast, (AROM+) mice showed increased production of estrogen and low levels of testosterone, resulting in the emergence of premalignant lesions and inflammation upon aging (42) [reviewed in (43)].

Considering these problems of administering estrogens or aromatase inhibitors, using SERMs may seem a better option in treating prostate cancer. SERMs can act as agonists or antagonists based on the tissue and cell types they act on (44). SERMs mostly act by modulating the interaction between the ER and co-regulatory proteins. The availability and concentration of the co-regulators vary between tissues and form the basis of the selective agonistic or antagonistic activity of the SERMs in different cell types [reviewed in (45)]. For example, cis-5,11-diethyl-5,6,11,12-tetrahydrochrysene-2,8-diol or THC, an R,R enantiomer, displays a contrasting effect on ER-α and ER-β transcriptional activity. Upon binding of THC to these receptors, they act as agonists toward the ER-α and antagonists against the ER-β. Tamoxifen, the first clinically approved SERM, is known to inhibit the proliferation in the PC3 and DU145 by inhibiting protein kinase C and inducing p21 (waf1/cip1) and in LNCaP cells in combination with mifepristone (46, 47). Tamoxifen along with quercetin inhibits angiogenesis and proliferation in CWR22 prostate tumor xenograft growth (48). These effects of SERMs highlight the importance of these molecules in the treatment of prostate cancer.

SERMs, such as tamoxifen, toremifene, and raloxifene, were shown to suppress prostate cancer cell proliferation in mouse models and cultured cells (47, 49–51). However, clinical trials did not show the same efficacy (52–54). However, the combination of toremifene and ADT in patients with advanced prostate cancer has been found to have a relatively better effect (55).

Raloxifene binds to both the ER-α and ER-β. In prostate cancer cell lines, it appears to selectively act on the ER-β and induce apoptosis (49, 50). In human-xenografted CWR22 and CWRSA9 cells, it appears to antagonize the ER-α and, hence, cause apoptosis (56).

The results obtained from prostate cancer cell lines and mouse models seem conflicting, and one of the major reasons is because of the differential expression of the ER-α and ER-β and the AR in these cells. Also, the cell line models do not take into consideration the tumor stroma which has an undeniable effect on the behavior of the tumor cells including the response to any drug (57). It has also been shown that ER-β-specific agonist in combination with AR targeting therapies decreases the survival of castrate-resistant prostate cancer (CRPC) cells (58). One of the possible mechanisms of this would be by activating the tumor suppressor PTEN (59).

Selective androgen receptor modulators (SARMs), similar to SERMs, bind and act on androgen receptors in a context-specific mechanism. SARMs have been used in prostate cancer treatment, but they have their own drawbacks. AR signaling has dual effects at different doses—inducing growth at lower activity levels while suppressing growth at higher levels. *In-vitro* and *in-vivo* studies have shown that SARMs repressed MYC oncoprotein expression and inhibited the growth of castration-sensitive and castration-resistant prostate cancer. However, further understanding is required for better efficacy (60). SARMs also have been used for treating triple-negative breast cancer cells, where they suppress the expression of genes and their associated pathways intratumorally, which encourages the development of breast cancer *via* its actions on the AR (61). For example, SARM RAD140 substantially inhibits the growth of AR/ER+ breast cancer patient-derived xenografts (PDX) by activating the AR and by suppressing ER-α action (62). However, this even requires further investigations.

Although this article is mainly focused on prostate cancer, considering that breast and prostate cancer are very similar in many aspects (6), some of the issues discussed here would be relevant in breast cancer as well. Hormone deprivation therapies such as androgen deprivation therapies, although they appear to be straightforward, have many complications in the long run, including the emergence of androgen independence. The differential distribution of estrogen receptors and the opposing effect of signaling in different cell types (such as epithelial/tumor- and stroma/cancer-associated fibroblasts) could be some of the causes of the emergence of androgen independence after administering ADT (**Figure 2**). Also, in some cases, it may be useful to activate hormonal signaling such as ER-β-mediated signaling to get a better effect (63). Hormones have very intricate feedback and feed forward regulations, because of which tampering with an intermediate in the pathway (Ex: Testosterone) can cause a major shift in the hormonal balance (Testosterone/Estrogen ratio) (**Figure S1**). A shift in the balance between androgen and estrogen due to localized alterations in biosynthesis and metabolism may result in unintentional adverse effects. It becomes extremely important to keep all these aspects in consideration while designing endocrine therapies so as to avoid endocrine resistance.

**Figure 2:**
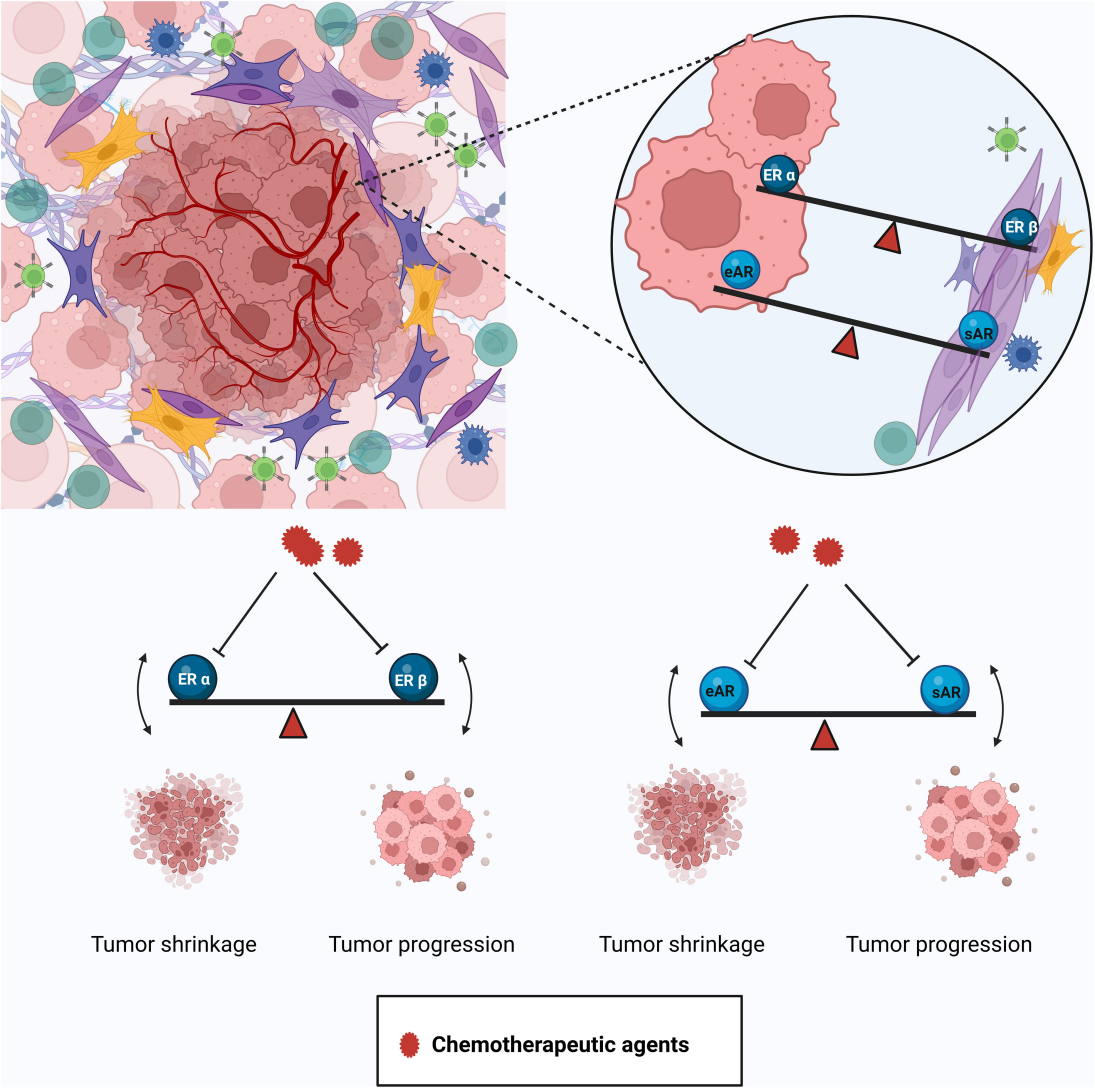
Differential distribution and effects of the AR and ER in the tumor and tumor microenvironment. The tumor is harbored in a complex microenvironment composed of different kinds of cells like immune cells, cancer-associated fibroblasts (CAFs), etc. The AR and ER show differential distribution and effects in the tumor and the stroma. Inhibiting the actions of the ER-α in the epithelium by certain drugs (e.g., tamoxifen) also suppresses the tumor-suppressive property of the ER-β in the stroma and disturbs the balance leading to resistant disease. Similarly, agents used to inhibit the tumor-promoting function of the epithelial AR (eAR) cause the stromal AR (sAR) to lose its tumor-suppressive action. Created in Biorender.com.

## Author contributions

AA collected the literature and helped prepare the manuscript. CS made the illustrations. PR conceptualized the article and wrote the manuscript. All authors contributed to the article and approved the submitted version.

## Supplementary material

Supplementary Figure 1 Schematic representation of steroid hormone synthesis in male and female mice.

## References

[1] Sever R Glass CK. Signaling by nuclear receptors. Cold Spring Harb Perspect Biol (2013) 5(3):a016709. doi: 10.1101/cshperspect.a016709 23457262 PMC3578364

[2] Alexandra R. SEX HORMONE SYNTHESIS, REGULATION, AND FUNCTION. Available at: http://www.pathophys.org/sexhormones.

[3] Ranganathan P Nadig N Nambiar S. Non-canonical estrogen signaling in endocrine resistance. Front Endocrinol (Lausanne) (2019) 10:708. doi: 10.3389/fendo.2019.00708 31749762 PMC6843063

[4] Vicent GP Nacht AS Zaurín R Ballaré C Clausell J Beato M. Minireview: Role of kinases and chromatin remodeling in progesterone signaling to chromatin. Mol Endocrinol (2010) 24(11):2088–98. doi: 10.1210/me.2010-0027 PMC5417384 20484412

[5] Castoria G Auricchio F Migliaccio A. Extranuclear partners of androgen receptor: At the crossroads of proliferation, migration, and neuritogenesis. FASEB J (2017) 31(4):1289–300. doi: 10.1096/fj.201601047R 28031322

[6] Risbridger GP Davis ID Birrell SN Tilley WD. Breast and prostate cancer: More similar than different. Nat Rev Cancer (2010) 10(3):205–12. doi: 10.1038/nrc2795 20147902

[7] Walker VR Korach KS. Estrogen receptor knockout mice as a model for endocrine research. ILAR J (2004) 45(4):455–61. doi: 10.1093/ilar.45.4.455 15454684

[8] Bocchinfuso WP Lindzey JK Hewitt SC Clark JA Myers PH Cooper R. Induction of mammary gland development in estrogen receptor-alpha knockout mice. Endocrinology (2000) 141(8):2982–94. doi: 10.1210/endo.141.8.7609 10919287

[9] Cooke PS Nanjappa MK Ko C Prins GS Hess RA. Estrogens in Male physiology. Physiol Rev (2017) 97(3):995–1043. doi: 10.1152/physrev.00018.2016 28539434 PMC6151497

[10] Chen M Yeh CR Chang HC Vitkus S Wen XQ Bhowmick NA. Loss of epithelial oestrogen receptor α inhibits oestrogen-stimulated prostate proliferation and squamous metaplasia *via in vivo* tissue selective knockout models. J Pathol (2012) 226(1):17–27. doi: 10.1002/path.2949 22069040 PMC3645347

[11] Rahman HP Hofland J Foster PA. In touch with your feminine side: How oestrogen metabolism impacts prostate cancer. Endocr Relat Cancer (2016) 23(6):R249–66. doi: 10.1530/ERC-16-0118 27194038

[12] Bonkhoff H. Estrogen receptor signaling in prostate cancer: Implications for carcinogenesis and tumor progression. Prostate (2018) 78(1):2–10. doi: 10.1002/pros.23446 29094395

[13] Yeh S Tsai MY Xu Q Mu XM Lardy H Huang KE. Generation and characterization of androgen receptor knockout (ARKO) mice: An *in vivo* model for the study of androgen functions in selective tissues. Proc Natl Acad Sci USA (2002) 99(21):13498–503. doi: 10.1073/pnas.212474399 PMC129702 12370412

[14] Schulster M Bernie AM Ramasamy R. The role of estradiol in male reproductive function. Asian J Androl (2016) 18(3):435–40. doi: 10.4103/1008-682X.173932 PMC4854098 26908066

[15] Ittmann M. Anatomy and histology of the human and murine prostate. Cold Spring Harb Perspect Med (2018) 8(5). doi: 10.1101/cshperspect.a030346 PMC5932577 29038334

[16] Rana K Davey RA Zajac JD. Human androgen deficiency: Insights gained from androgen receptor knockout mouse models. Asian J Androl (2014) 16(2):169–77. doi: 10.4103/1008-682X.122590 PMC3955325 24480924

[17] Cooper LA Page ST. Androgens and prostate disease. Asian J Androl (2014) 16(2):248–55. doi: 10.4103/1008-682X.122361 PMC3955334 24407178

[18] Chang C Lee SO Wang RS Yeh S Chang TM. Androgen receptor (AR) physiological roles in male and female reproductive systems: Lessons learned from AR-knockout mice lacking AR in selective cells. Biol Reprod (2013) 89(1):21. doi: 10.1095/biolreprod.113.109132 23782840 PMC4076350

[19] Simanainen U Allan CM Lim P McPherson S Jimenez M Zajac JD. Disruption of prostate epithelial androgen receptor impedes prostate lobe-specific growth and function. Endocrinology (2007) 148(5):2264–72. doi: 10.1210/en.2006-1223 17317769

[20] Wu CT Altuwaijri S Ricke WA Huang SP Yeh S Zhang C. Increased prostate cell proliferation and loss of cell differentiation in mice lacking prostate epithelial androgen receptor. Proc Natl Acad Sci USA (2007) 104(31):12679–84. doi: 10.1073/pnas.0704940104 PMC1937526 17652515

[21] Simanainen U McNamara K Gao YR McPherson S Desai R Jimenez M. Anterior prostate epithelial AR inactivation modifies estrogen receptor expression and increases estrogen sensitivity. Am J Physiol Endocrinol Metab (2011) 301(4):E727–35. doi: 10.1152/ajpendo.00580.2010 21750267

[22] Welsh M Moffat L McNeilly A Brownstein D Saunders PT Sharpe RM. Smooth muscle cell-specific knockout of androgen receptor: A new model for prostatic disease. Endocrinology (2011) 152(9):3541–51. doi: 10.1210/en.2011-0282 21733831

[23] Yu S Zhang C Lin CC Niu Y Lai KP Chang HC. Altered prostate epithelial development and IGF-1 signal in mice lacking the androgen receptor in stromal smooth muscle cells. Prostate (2011) 71(5):517–24. doi: 10.1002/pros.21264 PMC3037429 20945497

[24] Yu S Yeh CR Niu Y Chang HC Tsai YC Moses HL. Altered prostate epithelial development in mice lacking the androgen receptor in stromal fibroblasts. Prostate (2012) 72(4):437–49. doi: 10.1002/pros.21445 PMC4402036 21739465

[25] Liu Y Wang J Horton C Yu C Knudsen B Stefanson J. Stromal AR inhibits prostate tumor progression by restraining secretory luminal epithelial cells. Cell Rep (2022) 39(8):110848. doi: 10.1016/j.celrep.2022.110848 35613593 PMC9175887

[26] Richard RL John P. Oophorectomy for breast cancer: History revisited. J Natl Cancer Institute (2002) 94(19):1433–4. doi: 10.1093/jnci/94.19.1433 12359852

[27] Beatson GT. On the treatment of inoperable cases of carcinoma of the mamma: Suggestions for a new method of treatment, with illustrative cases. Trans Med Chir Soc Edinb (1896) 15:153–79. PMC5518378 29584099

[28] Haddow A Watkinson JM Paterson E Koller PC. Influence of synthetic oestrogens on advanced malignant disease. Br Med J (1944) 2(4368):393–8. doi: 10.1136/bmj.2.4368.393 PMC2286289 20785660

[29] Available at: https://www.cancer.net/cancer-types/breast-cancer/types-treatment#hormone-therapy.

[30] Huggins C Hodges CV. Studies on prostatic cancer. i. the effect of castration, of estrogen and of androgen injection on serum phosphatases in metastatic carcinoma of the prostate. 1941. J Urol (2002) 167(2 Pt 2):948–51. 11905923

[31] Cavalieri EL Stack DE Devanesan PD Todorovic R Dwivedy I Higginbotham S. Molecular origin of cancer: Catechol estrogen-3,4-quinones as endogenous tumor initiators. Proc Natl Acad Sci USA (1997) 94(20):10937–42. doi: 10.1073/pnas.94.20.10937 PMC23537 9380738

[32] Cavalieri E Frenkel K Liehr JG Rogan E Roy D. Estrogens as endogenous genotoxic agents–DNA adducts and mutations. J Natl Cancer Inst Monogr (2000) (27):75–93. doi: 10.1093/oxfordjournals.jncimonographs.a024247 10963621

[33] Cavalieri EL Devanesan P Bosland MC Badawi AF Rogan EG. Catechol estrogen metabolites and conjugates in different regions of the prostate of noble rats treated with 4-hydroxyestradiol: Implications for estrogen-induced initiation of prostate cancer. Carcinogenesis (2002) 23(2):329–33. doi: 10.1093/carcin/23.2.329 11872641

[34] Jefcoate CR Liehr JG Santen RJ Sutter TR Yager JD Yue W. Tissue-specific synthesis and oxidative metabolism of estrogens. J Natl Cancer Inst Monogr (2000) 27:95–112. doi: 10.1093/oxfordjournals.jncimonographs.a024248 10963622

[35] Han X Liehr JG Bosland MC. Induction of a DNA adduct detectable by 32P-postlabeling in the dorsolateral prostate of NBL/Cr rats treated with estradiol-17 beta and testosterone. Carcinogenesis (1995) 16(4):951–4. doi: 10.1093/carcin/16.4.951 7728979

[36] Vihko P Herrala A Härkönen P Isomaa V Kaija H Kurkela R. Control of cell proliferation by steroids: The role of 17HSDs. Mol Cell Endocrinol (2006) 248(1-2):141–8. doi: 10.1016/j.mce.2005.12.005 16406264

[37] Carruba G Adamski J Calabrò M Miceli MD Cataliotti A Bellavia V. Molecular expression of 17 beta hydroxysteroid dehydrogenase types in relation to their activity in intact human prostate cancer cells. Mol Cell Endocrinol (1997) 131(1):51–7. doi: 10.1016/S0303-7207(97)00092-0 9256363

[38] Fisher CR Graves KH Parlow AF Simpson ER. Characterization of mice deficient in aromatase (ArKO) because of targeted disruption of the cyp19 gene. Proc Natl Acad Sci USA (1998) 95(12):6965–70. doi: 10.1073/pnas.95.12.6965 PMC22703 9618522

[39] Jones ME Thorburn AW Britt KL Hewitt KN Wreford NG Proietto J. Aromatase-deficient (ArKO) mice have a phenotype of increased adiposity. Proc Natl Acad Sci U S A (2000) 97(23):12735–40. doi: 10.1073/pnas.97.23.12735 PMC18833 11070087

[40] Honda S Harada N Ito S Takagi Y Maeda S. Disruption of sexual behavior in male aromatase-deficient mice lacking exons 1 and 2 of the cyp19 gene. Biochem Biophys Res Commun (1998) 252(2):445–9. doi: 10.1006/bbrc.1998.9672 9826549

[41] Evan RS Margaret EJ Colin DC. Lessons from the ArKO mouse. In: F. BJA, editor. Aromatase inhibitors. Basel, Switzerland: Milestones in Drug Therapy (2006). p. 139–55.

[42] Ellem SJ Risbridger GP. Aromatase and regulating the estrogen:androgen ratio in the prostate gland. J Steroid Biochem Mol Biol (2010) 118(4-5):246–51. doi: 10.1016/j.jsbmb.2009.10.015 19896534

[43] Ellem SJ Risbridger GP. Aromatase and prostate cancer. Minerva Endocrinol (2006) 31(1):1–12. 16498360

[44] Jordan VC. Selective estrogen receptor modulation: Concept and consequences in cancer. Cancer Cell (2004) 5(3):207–13. doi: 10.1016/S1535-6108(04)00059-5 15050912

[45] Dutertre M Smith CL. Molecular mechanisms of selective estrogen receptor modulator (SERM) action. J Pharmacol Exp Ther (2000) 295(2):431–7. 11046073

[46] Rohlff C Blagosklonny MV Kyle E Kesari A Kim IY Zelner DJ. Prostate cancer cell growth inhibition by tamoxifen is associated with inhibition of protein kinase c and induction of p21(waf1/cip1). Prostate (1998) 37(1):51–9. doi: 10.1002/(SICI)1097-0045(19980915)37:1<51::AID-PROS8>3.0.CO;2-B 9721069

[47] El Etreby MF Liang Y Lewis RW. Induction of apoptosis by mifepristone and tamoxifen in human LNCaP prostate cancer cells in culture. Prostate (2000) 43(1):31–42. doi: 10.1002/(SICI)1097-0045(20000401)43:1<31::AID-PROS5>3.0.CO;2-# 10725863

[48] Ma ZS Huynh TH Ng CP Do PT Nguyen TH Huynh H. Reduction of CWR22 prostate tumor xenograft growth by combined tamoxifen-quercetin treatment is associated with inhibition of angiogenesis and cellular proliferation. Int J Oncol (2004) 24(5):1297–304. doi: 10.3892/ijo.24.5.1297 15067354

[49] Kim IY Kim BC Seong DH Lee DK Seo JM Hong YJ. Raloxifene, a mixed estrogen agonist/antagonist, induces apoptosis in androgen-independent human prostate cancer cell lines. Cancer Res (2002) 62(18):5365–9. 12235008

[50] Kim IY Seong DH Kim BC Lee DK Remaley AT Leach F. Raloxifene, a selective estrogen receptor modulator, induces apoptosis in androgen-responsive human prostate cancer cell line LNCaP through an androgen-independent pathway. Cancer Res (2002) 62(13):3649–53. 12097269

[51] Raghow S Hooshdaran MZ Katiyar S Steiner MS. Toremifene prevents prostate cancer in the transgenic adenocarcinoma of mouse prostate model. Cancer Res (2002) 62(5):1370–6. 11888907

[52] Bergan RC Reed E Myers CE Headlee D Brawley O Cho HK. A phase II study of high-dose tamoxifen in patients with hormone-refractory prostate cancer. Clin Cancer Res (1999) 5(9):2366–73. 10499606

[53] Stein S Zoltick B Peacock T Holroyde C Haller D Armstead B. Phase II trial of toremifene in androgen-independent prostate cancer: A Penn cancer clinical trials group trial. Am J Clin Oncol (2001) 24(3):283–5. doi: 10.1097/00000421-200106000-00015 11404501

[54] Price D Stein B Sieber P Tutrone R Bailen J Goluboff E. Toremifene for the prevention of prostate cancer in men with high grade prostatic intraepithelial neoplasia: Results of a double-blind, placebo controlled, phase IIB clinical trial. J Urol (2006) 176(3):965–70. doi: 10.1016/j.juro.2006.04.011 16890670

[55] Fujimura T Takahashi S Kume H Urano T Takayama K Yamada Y. Toremifene, a selective estrogen receptor modulator, significantly improved biochemical recurrence in bone metastatic prostate cancer: A randomized controlled phase II a trial. BMC Cancer (2015) 15:836. doi: 10.1186/s12885-015-1871-z 26526623 PMC4630884

[56] Shazer RL Jain A Galkin AV Cinman N Nguyen KN Natale RB. Raloxifene, an oestrogen-receptor-beta-targeted therapy, inhibits androgen-independent prostate cancer growth: Results from preclinical studies and a pilot phase II clinical trial. BJU Int (2006) 97(4):691–7. doi: 10.1111/j.1464-410X.2006.05974.x 16536755

[57] Di Donato M Giovannelli P Cernera G Di Santi A Marino I Bilancio A. Non-genomic androgen action regulates proliferative/migratory signaling in stromal cells. Front Endocrinol (Lausanne) (2014) 5:225. doi: 10.3389/fendo.2014.00225 25646090 PMC4298220

[58] Gehrig J Kaulfuß S Jarry H Bremmer F Stettner M Burfeind P. Prospects of estrogen receptor β activation in the treatment of castration-resistant prostate cancer. Oncotarget (2017) 8(21):34971–9. doi: 10.18632/oncotarget.16496 PMC5471027 28380417

[59] Wu WF Maneix L Insunza J Nalvarte I Antonson P Kere J. Estrogen receptor β, a regulator of androgen receptor signaling in the mouse ventral prostate. Proc Natl Acad Sci U S A (2017) 114(19):E3816–22. doi: 10.1073/pnas.1702211114 PMC5441728 28439009

[60] Nyquist MD Ang LS Corella A Coleman IM Meers MP Christiani AJ. Selective androgen receptor modulators activate the canonical prostate cancer androgen receptor program and repress cancer growth. J Clin Invest (2021) 131(10). doi: 10.1172/JCI146777 PMC8121509 33998604

[61] Narayanan R Ahn S Cheney MD Yepuru M Miller DD Steiner MS. Selective androgen receptor modulators (SARMs) negatively regulate triple-negative breast cancer growth and epithelial:mesenchymal stem cell signaling. PLoS One (2014) 9(7):e103202. doi: 10.1371/journal.pone.0103202 25072326 PMC4114483

[62] Yu Z He S Wang D Patel HK Miller CP Brown JL. Selective androgen receptor modulator RAD140 inhibits the growth of Androgen/Estrogen receptor-positive breast cancer models with a distinct mechanism of action. Clin Cancer Res (2017) 23(24):7608–20. doi: 10.1158/1078-0432.CCR-17-0670 28974548

[63] Paterni I Granchi C Katzenellenbogen JA Minutolo F. Estrogen receptors alpha (ERα) and beta (ERβ): Subtype-selective ligands and clinical potential. Steroids (2014) 90:13–29. doi: 10.1016/j.steroids.2014.06.012 24971815 PMC4192010

