# Supplementary figures and images for: To block or not to block—hormonal signaling in the treatment of cancers
Source: Front Endocrinol (Lausanne). 2023 Feb 20;14:1129332. doi: 10.3389/fendo.2023.1129332 (PMC9986485; doi:10.3389/fendo.2023.1129332)

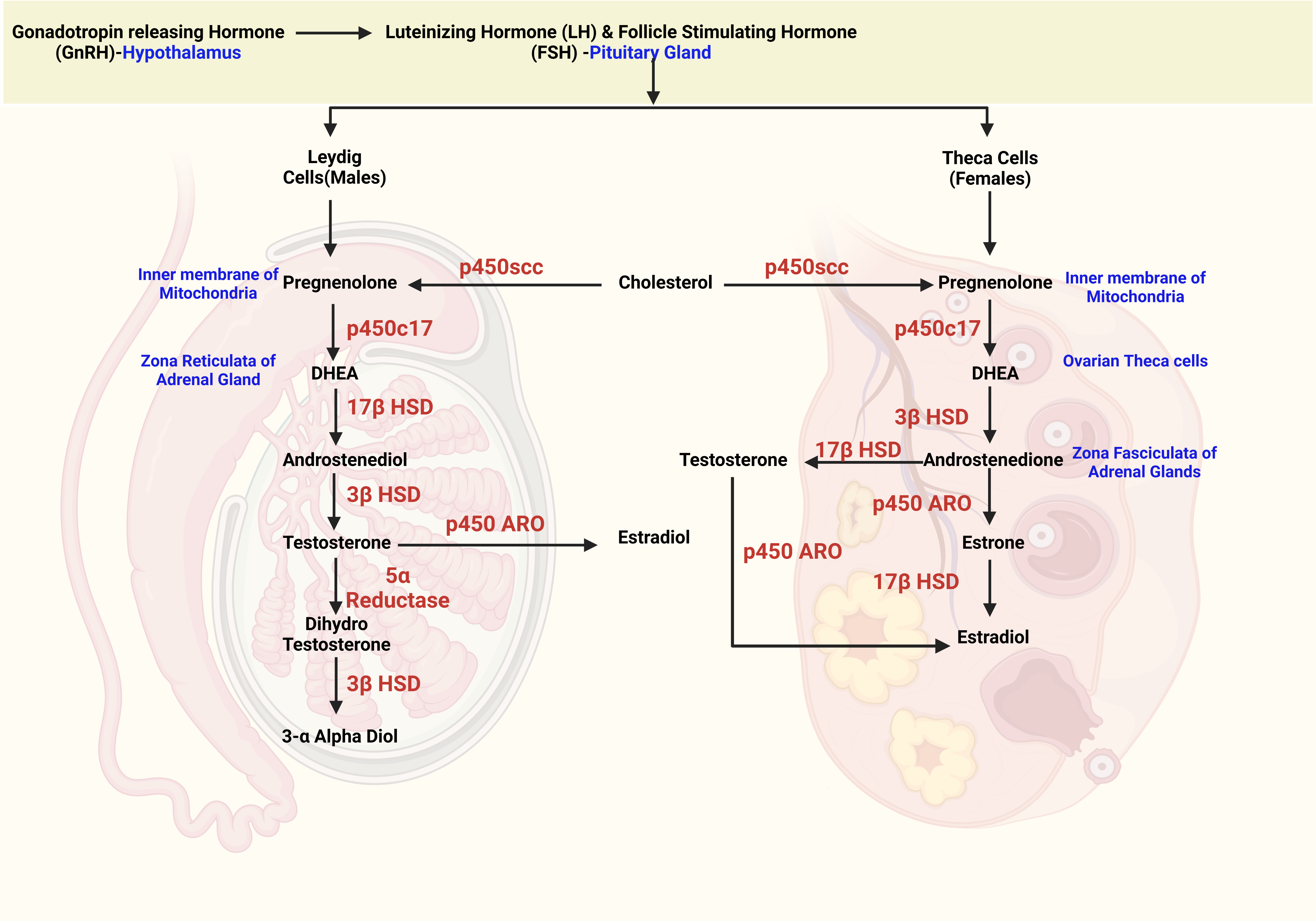

Supplement: Supplementary Figure 1 — Schematic representation of steroid hormone synthesis in male and female mice. [file Image_1.jpg]
